# Urinary schistosomiasis among preschool children in a rural community near Abeokuta, Nigeria

**DOI:** 10.1186/1756-3305-3-58

**Published:** 2010-07-05

**Authors:** Uwem F Ekpo, Akintunde Laja-Deile, Akinola S Oluwole, Sammy O Sam-Wobo, Chiedu F Mafiana

**Affiliations:** 1 Department of Biological Sciences, University of Agriculture, Abeokuta, Nigeria; 2 Office of the Executive Secretary, National Universities Commission, Abuja, Nigeria

## Abstract

**Background:**

The control of schistosomiasis in Nigeria is mainly by mass treatment with praziquantel through the school system, with an absence of any provision for pre-school children. We therefore determined the prevalence and intensity of urinary schistosomiasis in pre-school children between the ages of 1-6 years in Ilewo-Orile a rural and endemic community, near Abeokuta, Nigeria as part of providing information on the neglected tropical diseases among this age group. Two urine samples were collected from each pre-school child. The samples were tested for microhaematuria using reagent strips and then processed and examined with a microscope for *Schistosoma haematobium *ova.

**Results:**

Of the 167 children examined 97 (58.1%) had infection, with no significant difference (P = 0.809) in infection rates between boys (57.1%) and girls (59.2%). Both prevalence and intensity of infection did not increase significantly with age (P = 0.732). The overall geometric mean egg count was 1.17 eggs/10 ml urine. There was no significant association (*P *= 0.387) between intensity in boys (1.16 eggs/10 ml urine) and girls (1.19 eggs/10 ml urine). 47.4% of the children had microhaematuria which did not increase significantly with age (P = 0.526). Focus group discussions with guardians and caregivers revealed that infection of pre-school children early in life was due to exposures through bathing in the stream by their mothers, while the older children would visit the stream for washing, fetching of water, bathing and swimming.

**Conclusion:**

Community participatory health education is needed in this community as a first step in reducing infection and transmission of the disease, while the rehabilitation and repair of the existing water borehole system in the community should be effected. The results of this study have shown that pre-school children also harbour infection and are a source of transmission of schistosomiasis in endemic communities. Planning and provision for their treatment should be considered in control programmes.

## Background

Urinary schistosomiasis is a human disease condition, which is caused by infection of the trematode *Schistosoma heamatobium*. The parasite is found in the venous plexus draining the urinary bladder of humans [1]. During infection, the parasites deposit terminal spined eggs which clog the venous plexus, impeding blood flow. This bursts the veins, allowing blood and eggs to enter the urinary bladder, resulting in the characteristic symptom of blood in urine or haematuria [1]. In sub-Saharan Africa alone it is estimated that 70 million individuals experience heamaturia, 32 million with difficulty in urinating (dysuria), 18 million with bladder-wall pathology, and 10 million with major hydronephrosis from infection caused by *Schistosoma haematobium*. Mortality rate due to non-functioning kidney (from *S. haematobium*) and haematemesis has been put at 150,000 per year [2]. The above figures imply that urinary schistosomiasis is an important public health problem in sub-Saharan Africa and second to malaria in morbidity [3].

Urinary schistosomiasis is endemic in Nigeria in general [1]. Although there is no current estimate of the disease in the country, past estimates have put the infection at about 25 million people and 101 million at risk of infection respectively [4]. In Ogun State, urinary schistosomiasis has been reported in several communities [5-11]. However, these studies were based on school-aged children and adults with little or no information on pre-school children. Recently, urinary schistosomiasis infections have been reported in pre-school children in settlements near Oyan dam, Ogun State and Adim, Cross River State, all in Nigeria [12,13]. Even then, there is still the need to obtain more information on schistosomiasis in this age group in order to justify their inclusion in mass treatment programmes.

## Results

A total of 213 pre-school children were enrolled for this study, but only 167 children submitted urine samples for analysis. The ages of the pre-school children ranged from 1-6 years with a mean of 3.51 years. The 3 year-olds included the highest number of pre-school children 34 (20.4%). Ninety-one (54.5%) of them were boys while 76 (45.5%) were girls.

### Prevalence and intensity of *S. heamatobium *infection by age and sex

A total of 97 (58.1%) children tested positive *S. haematobium *infection. Pre-school children of 4 years old had the highest occurrence with 18 (69.2%) children. The least occurrence was among those aged 1 year (52.4%). However, there was no significant difference in infection between ages (P = 0.732). Likewise there were no significant differences (P = 0.787) in prevalence between the girls 45 (59.2%) the boys 52 (57.1%). The geometric mean intensity of infection was 1.17 eggs/10 ml of urine. There was no significant differences (p = 0.387) in intensity of infection between the boys (1.15 eggs/10 ml) and girls (1.19 eggs/10 ml). Pre-school children of 3 years of age had the highest mean intensity of infection of 1.34 eggs/10 ml while the least of 0.96 eggs/10 ml was in pre-school children of 1 year old. However there was no significant differences (p = 0.087) between age and intensity of infection (Table 1).

**Tabel 1:** Prevalence and intensity of *Schistosoma haematobium *infection and microhaematuria by age and sex of preschool children in Ilewo Orile community

	No. Examined	No and % infected	No (%) with microhaematuria	Geometric Mean Intensity of infection
Total	167	97 (58.1)	79 (47.4)	1.17

				

Sex				

Boys	91	52 (57.1)	43 (25.8)	1.15

Girls	76	45 (59.2)	36 (21.6)	1.19

P value		0.787	0.562	0.387

				

Age				

1 year old	21	11(52.4)	7 (4.2)	0.96

2 years old	32	18 (56.3)	14(8.4)	1.16

3 years old	34	17 (50.0)	15 (9,0)	1/34

4 years old	26	18 (69.2)	12 (7.2)	1.06

5 years old	29	18 (62.1)	16 (9.6)	1.21

6 years old	25	15 (60.0)	15 (9.0)	1.24

P value		0.732	0.942	0.087

The prevalence of micohaematuria by age showed that a total of 79 (47.4%) children tested positive for microhaematuria in their urine of which those of age 5 years old had the highest prevalence with 16 (9.6%). However, there was no significant difference between microhaematuria and age (P = 0.526). The boys 43 (25.8%) had more of microhaematuria than girls but there was no significant difference in the occurrence of microhaematuria between the sexes (P = 0.942) (Table 1).

### Water contact practices

The community heads helped in the mobilization of parents and guardians into three groups in which the water contact practices was fully discussed. The focus group discussions (FGDs) with guardians and caregivers in the community showed that Owi River was the only source of water supply in the community. As such, the river served as the source of water for bathing, drinking, washing, recreation and cooking. The water bore-hole system installed by government in the community is no longer functioning. Guardians and caregivers with pre-school children acknowledged that they usually visit the river to bathe their children or bring them along to assist in washing clothes or kitchen utensils. Whereas pre-school children between the ages of 4-6 years old admitted they visit the river every day for washing, fetching of water, bathing and swimming. Members of the community are aware that the symptom of passing blood in the urine called "*Atosi aja*" the local name for urinary schistosomiasis however, they do not consider their river as the source of infection. They believed that the disease is only a sign of virility and coming to adulthood, which is also common in other communities near them. They ignorantly submitted that any infection that is contacted through water and penetration of the skin must show on the skin surface and not in their urine. They submitted that urinary schistosomiasis has existed in their community for many years and that they do not consider it as their major health problem when compared to malaria fever. When asked about the non-functional borehole in the community, they mentioned that the borehole mainly provided them with water for drinking and since it has stopped functioning, they have no other alternative but to return to the river, which is always available.

## Discussion

Several studies of urinary schistosomiasis have tended to focus on school-age children and adults, with little or no emphasis on pre-school children, and where pre-school children are part of the study, information about them was often subsumed [14,15]. The result of this study shows a prevalence rate of 58.1% of urinary schistosomiasis among the pre-school children in Ilewo-Orile community. This result is comparable to 71.8% in settlements near a dam reservoir, Ogun State, Nigeria and 19.8% in a rice farming community of Adim, Cross River State, Nigeria [12,13]. The differences in prevalence among these studies could be attributed to types of water bodies and water contact practices which need further investigation. All the ages studied had infection, meaning that infection with urinary schistosomiasis occurs very early in life through exposure to contaminated water bodies either by guardians and caregivers or the pre-school children themselves. As the stream is the only source of water supply in the community, it difficult if not impossible to prevent the community from visiting this stream everyday for various uses.

However, there was no significant difference in the prevalence between boys (57.1%) and girls (59.2%) as well as age. This may be an indication that both gender and ages are equally exposed to infection through water contacts. Contacts with the stream by pre-school children remain unabated throughout their pre-school age. The mean intensity of infection reported here is lower than that of [13]. The reason for this could be due to several factors such as immunity, water contact practices and density of infected snail intermediate hosts in the water bodies.

The prevalence of microhaematuria in this study (47.4%) shows no significant difference between the age and sex (P > 0.05). This is due to the fact that microhaematuria is a characteristic symptom of urinary schistosomiasis in endemic communities where its prevalence correlated positively with urinary schistosomiasis infection [16].

The relatively high prevalence (58.1%) of this study calls for community mobilisation and participation in the control of urinary schistosomiasis in Ilewo Orile. Past efforts to control the disease through the provision of a water-borehole has failed. Apart from the fact the borehole is not functioning, the community members will only use the borehole as a drinking water source. Therefore, for any control measure that are to succeed here must involve the community [17], such as developing participatory health education programmes with community members to understand disease transmission, effect behavioural change by mothers who expose their children to the infection. Involving the community in repairing their safe water source could help reduce the contact with stream water and transmission of the disease. The mothers of the children should also be enlightened about the avoidance of the stream water with their children by heating the water before using it to bathe and not taking the children along with them to the stream [12].

There have been several calls for inclusion of infant and pre-school children for treatment in schistosomiasis control programmes in endemic countries [12,13,18-21]. Although, we treated all pre-school children that tested positive for schistosomiasis with the WHO standard recommended dose 40 mg/kg of praziquantel, there were no apparent side effects or non-compliance issues, as parents and care givers were happy to have their children treated as there is no ongoing treatment programme in the State. However, the issue of the safety of praziquantel in infants less than 3 years old needs to be addressed as seen in a recent study where side effects were reported [22]. Therefore, further studies are need in other endemic settings on the safety of praziquantel treatment in infants so that the treatment can be extended to this age group.

## Conclusion

The emerging fact from the study shows that pre-school children also harbour infection with urinary schistosomiasis, and are therefore also source of transmission in endemic communities. Although currently excluded from mass treatment programmes, provision for their inclusion in treatment programmes is imperative and should be considered.

## Materials and methods

### Study area

Ilewo-Orile is a rural community (latitude 7.13333N and longitude 3.18333E) and approximately 20 kilometres from Abeokuta, the State capital of Ogun State, Southwest Nigeria. Members of this community are farmers and traders of the Yoruba ethnic group. Passing through the community is a flowing stream called Owi River which is the major source of water in the community as the water bore-hole system in the community is non-functional. The community has no health centre. A total of 213 pre-school children were enumerated in the community in a house-to-house census conducted for children below 6 years of age. Children were registered using an epidemiological form which documented their age, sex, and the name of the household head. Due to the small number of pre-school children in the community, sampling and sample size calculation were not necessary. However, only 167 pre-school children provided urine samples for examination.

### Urine Collection

Dark (black), sterile, plastic universal containers (labelled) were given to the parents/guardians of the children to collect urine samples. This was done between the hours of 10.00 am to 2.00 pm. The urine collected was then immediately taken to the laboratory for analysis. A total of 167 children had their samples returned and these were subsequently used for the study.

### Examination of Urine for Microhaematuria

A reagent strip (Urine-10 parameters, Cyress Diagnostics (3201 Langdorp-Belgium) was carefully dipped into the dark sterile bottle containing the urine for 5 seconds. The resulting change in colour of the strip was compared with manufacturer's colour chart to estimate the amount of blood in the urine.

### Examination for *Schistosoma haematobium *ova

10 ml of the urine sample was centrifuged at 5000 rpm for 5 minutes. The supernatant was discarded to leave sediment which was transferred to the centre of a clean grease-free glass slide to which was added a cover slip. This was mounted on a light microscope and examined at ×40 objective to identify *Schistosoma haematobium *ova which is characterised with a terminal spine. The eggs were counted and recorded as eggs/10 ml of urine. Those positive for urinary schistosomiasis infection were treated with 40 mg/kg of praziquantel.

### Focus Group Discussion

Focus Group Discussions (FGDs) on perceptions of the disease was held in the community with guardians, care givers and pre-school children, using an interview guide. There were three sessions each with pre-school children, adult males and adult females (mostly nursing mothers). The sessions with pre-school children were limited to children between 4 to 6 years of age. Each discussion group comprised a minimum of six and maximum of ten people randomly selected from willingly participants. In addition an in-depth interview concerning perceptions of urinary schistosomiasis, water contact practices and disease control activities was held with the village leader. A portable electronic voice recorder was used to record the discussions.

### Data Analysis

The data were entered and analysed using SPSS version 16.0 for Windows. Differences in proportions were tested using chi-square test, either for trend or for independence, as appropriate. The number of eggs counted were transformed to log10 (x+1) values to normalize the distribution of the residuals values for statistical analyses. Differences between means were tested using independent samples t-test and one way ANOVA. Qualitative data from the FGDs and in-depth interview were transcribed and analysed manually.

### Ethical approval

Before the study began, the village head together with guardians and caregivers were fully briefed on the objective of the study. Thereafter, the guardians and caregivers were given an informed consent form to sign for themselves and their wards, after its content was translated to them in local language. Only pre-school children whose guardian and caregivers signed the consent form participated in the study. The study objective was also explained to older preschool children for their understanding and cooperation. The study protocol was reviewed and approved by the Ogun State Ministry of Health, and the ethical review board of the College of Natural Sciences, University of Agriculture, Abeokuta.

## Competing interests

The authors declare that they have no competing interests.

## Authors' contributions

UFE and ALD initiated the study, UFE and ALD designed the study. ALD collected the data and UFE, ASO, and SOSW supervised the data collection. ASO, ALD and UFE did the data analysis and interpretation; UFE, ALD and ASO wrote the manuscript. UFE, ALD, ASO, SOSW and CFM reviewed the manuscript. All authors read and approved the final manuscript.
